# Galectin-Levels Are Elevated in Infants Born Preterm Due to Amniotic Infection and Rapidly Decline in the Neonatal Period

**DOI:** 10.3389/fimmu.2020.599104

**Published:** 2021-02-25

**Authors:** Kirstin Faust, Nancy Freitag, Gabriela Barrientos, Christoph Hartel, Sandra M. Blois

**Affiliations:** ^1^Department of Pediatrics, University of Luebeck, University Hospital of Schleswig-Holstein, Lübeck, Germany; ^2^German Center for Infection Research, Lübeck, Germany; ^3^Department of Obstetrics and Fetal Medicine, University Medical Center Hamburg-Eppendorf, Hamburg, Germany; ^4^Experimental and Clinical Research Center, Max Delbrück Center for Molecular Medicine in the Helmholtz Association, Charité—Universitätsmedizin Berlin, Berlin, Germany; ^5^Charité-Universitätsmedizin Berlin, Division of General Internal and Psychosomatic Medicine, Berlin, Germany; ^6^Laboratorio de Medicina Experimental, Hospital Alemán-Consejo Nacional de Investigaciones Científicas y Técnicas, Buenos Aires, Argentina; ^7^Department of Paediatrics, University of Würzburg, Würzburg, Germany

**Keywords:** galectin-1, galectin-3, galectin-9, preterm infants, amniotic infection

## Abstract

Galectin (gal)-1, -3, and -9 are members of a family of glycan binding proteins that mediate complex interactions between decidual, inflammatory and trophoblast cells modulating several processes during gestation, control of the maternal immune system, and parturition. Their immunomodulatory role in preterm birth and postnatal expression in preterm infants is unknown. We performed a single center prospective study of 170 preterm infants with a gestational age below 35 weeks. Peripheral venous blood samples were collected during the neonatal period and galectin-1, -3, and -9 were determined by ELISA. We noted a strong decline of circulating gal-1 and -3 levels but not gal-9 from birth to day 7 of life. There was an inverse correlation of gal-1 and -3 levels at birth with gestational age. Gal-1 levels were remarkably increased in infants born to amniotic infection syndrome (AIS), which was also observed for gal-9 levels. Infants who developed early-onset sepsis had higher levels of gal-3 at day 1 as compared to unaffected infants. Our observational data imply that galectin-1, -3, and -9 levels are elevated in preterm infants born in an inflammatory milieu such as AIS or EOS. Future studies need to address whether galectins mediate inflammation-induced preterm birth and could therefore be a target for clinical trials.

## Introduction

Preterm birth remains an unresolved health problem, accounting worldwide for an approximate 10% of live births. Preterm infants are predisposed to sepsis due to vulnerable body surfaces, immature immunity, and a variety of injurious exposures, particularly in the first 4 weeks of life. This is a continuing challenge to clinicians involved in the care of preterm infants, as sepsis and sustained inflammation are regarded as crucial mediators for mortality and the development of long-term morbidities (1). Hence there is an urgent need to identify risk factors and biomarkers aiming at prevention and early treatment of sepsis.

Galectins, a family of glycan binding proteins, are multifactorial regulators of pregnancy associated process including cellular interactions between decidual, inflammatory, and trophoblast cells during gestation and parturition (2). Based on their structure, galectins are classified into three different types (3): 1) prototype [e.g., galectin- 1 (gal-1)], containing one carbohydrate recognition domain (CRD) per subunit that can dimerize, 2) chimera-type (gal-3), with a carboxyl-terminal CRD joined to an amino-terminal peptide allowing pentamer formation, and 3) tandem-repeat (gal-9), displaying two CRD joined by a functional linker peptide. Their main functions during gestation is the control of the maternal immune system, regulation of placental development, and expansion of the maternal-placental vasculature (4–6). Although data on galectins and parturition is relatively scarce, a dysregulation of the glycan binding proteins has been discussed (2). Gal-1 is the most abundant galectin in the human decidua at term, however its decidual expression and that of gal-3 decreased as laboring progressed (7). Maternal gal-9 circulating levels are elevated at the time of parturition but returning to non-pregnant levels during postpartum (8). Consistent with this, the chimera lectin, gal-3 is highly expressed at the decidual and placental compartment and after parturition its expression dramatically decreased as the implantations sites resorbed (9, 10). Although galectin interactions during gestation are well characterized, the potential outcome of these interactions in the context of preterm birth are poorly defined. For instance, a transcriptomic and proteomic profiling comparing changes in choriodecidual tissue from women who suffered from spontaneous preterm labor and gestational-matched non-laboring controls has identified gal-1 to be upregulated only during preterm labor, suggesting a possible association with the underlying pathology (11). In this context, gal-1 expressed on decidual cells has been shown to down-regulate the IL-6 induced by LPS stimulus via NF-κB signal pathway inhibition (12, 13). As IL-6 is an important cytokine related to PTB, gal-1 may be important in the regulation of local inflammation during the course of chorioamnionitis (13). Moreover, gal-3 expression increased in fetal membranes and in the amniotic epithelium in patients with chorioamniotic infection (14), thereby regulating the inflammatory response and/or direct interaction with the pathogens.

In the context of preterm birth, levels of gal-1 and gal-3 in cord blood correlated with gestational age, growth restriction and maternal diabetes (15, 16). Decreased cord blood levels of gal-3 binding protein were noted in association with chorioamnionitis (17) or in infants with later development of chronic lung disease (18). The understanding of the potential immunomodulatory effects of galectins in preterm infants is crucial. The present study characterizes the postnatal dynamics of gal-1, -3, and -9 circulating levels in a well-phenotyped cohort of preterm infants with particular focus on an “inflammatory” environment.

## Material and Methods

### Study Population

We performed a prospective single-center study in the perinatal unit of the Department of Obstetrics and Gynaecology at the University of Luebeck, Germany. We enrolled a convenience sample of 170 preterm infants with a gestational age below 35 weeks. Infants with lethal abnormalities were excluded. This study was part of our IRoN (Immunoregulation of the Newborn) study project.

### Ethical Approval

Ethical approval was given for all study parts by the University of Luebeck Ethical Committee (IRON AZ 15-304). Informed written consent was given by parents (as legal representatives) on behalf of their infants. All blood samples were obtained within a medically required blood withdrawal procedure.

### Definitions

Data were collected from clinical records of mother and child. Gestational age was defined as best estimate according to post-menstrual age (obstetrical dating). Amniotic infection (AIS) was diagnosed when more than two of the following clinical signs were noted: maternal fever (temperature > 38.0°C) or fetal tachycardia, maternal elevation of white blood cell count >10/nL or c-reactive protein levels (> 20 mg/L) without other focus of infection, painful uterus, foul-smelling amniotic fluid, preterm labor, preterm rupture of membranes or early-onset sepsis of the newborn infant. Reasonable suspicion for AIS was assumed if only one of the signs was noted. Other reasons for delivery included preeclampsia, pathological vascular Doppler or pathological cardiotocography, placental abruption and others.

Small for gestational age (SGA) was defined as a birth weight less than 10^th^ percentile according to gender-specific standards for birth weight by post menstrual age in Germany (19). Sepsis was diagnosed using the NEO-KISS criteria as clinical sepsis or blood-culture proven sepsis (20, 21). Early-onset sepsis (EOS) was defined as sepsis (clinical or blood culture pos.) occurring within the first 72 h of life. Late-onset sepsis (LOS) was defined as sepsis (clinical or blood culture pos.) occurring after the first 72 h of life but before 25 days of life.

### Collection of Blood Samples

Peripheral venous blood samples were collected along with routine laboratory sampling at day 1, 3, 7, and 21 and 28 of life in tubes containing 16 I.E. heparin/ml. Plasma samples were obtained after centrifugation (6 min, 3,000 g) and stored at -20°C until analysis. Maximum storage time before centrifugation was 24 h.

### Galectin-1, -3, and -9 ELISA

Gal-1, -3, or -9 concentrations in plasma samples were determined by ELISA as described previously (4, 22). Briefly, Corning^®^ 96-Well High-Binding (Fisher-scientific) were covered with polyclonal anti human gal-1, or gal-3 or gal-9 antibodies (500-P210 Petrotech, AF1154 and AF 2045; R&D Systems, USA respectively) and washed with washing buffer (0.5% Tween-20 in PBS). Plates were blocked with 3% BSA in PBS. Individual wells were incubated with serial dilutions of gal-1, -3, or -9 or plasma samples (diluted 1/4) for 2 h at RT. Wells were washed and incubated with biotinylated polyclonal anti-human gal-1, or gal-3 or gal-9 antibodies (500-P210BT Petrotech, BAF1154, BAF2045 R&D Systems respectively). Plates were washed six times and incubated with horseradish peroxidase (HRP)-conjugated streptavidin (Calbiochem, USA). After eight additional washes, a colorimetric reaction was developed with the 3,3,5,5′-tetramethyl benzidine (TMB) substrate (Pierce Biotechnology, USA). The reaction was stopped by adding one volume of 4 N H2SO4. Absorbance at 450 nm was recorded. Each reported value is the mean of duplicate assays.

### Statistical Analysis

Mann-Whitney U-test and Kruskal-Wallis-Test were applied for statistical analysis of differences between two or three non-paired groups, correlations were tested by the Spearman-Rho test. The level of significance was defined as p <0.05 in single comparisons and adjusted with Bonferroni-correction for multivariable testing. For single comparisons, the postnatal age was assessed in groups (based on the five time points of sample collection), for multivariable testing the data was transformed using postnatal age in days as a continuous variable.

We used multivariable linear regression for assessment of the combined influence of gestational age and postnatal age (dependent variable: Galectin level, independent variables: gestational age, birth weight, and postnatal age).

Generalized estimating equation (GEE) with gaussian error and autoregressive [AR(1)] correlation matrix was used to determine the association between the gal levels measured on different days of life and the interaction effects between the timepoint of measurement (day of life) and either gestational age, AIS, or EOS. All variables were time-invariant except for gal levels and day of life. Statistical analysis was performed using SPSS_25.0 statistical software (SPSS Inc., Chicago, USA).

## Results

### Clinical Characteristics of Infants

**Table 1** and **Supplementary Table 1** describe the clinical characteristics of the cohort (n = 170) with a mean (± SD) gestational age of 28.7 ± 3.1 weeks (GA) and birth weight of 1121 ± 449 g. The cohort included 62 multiples (36.5%), 93 (54.7%) male, and 38 (22.4%) SGA (small for gestational age) infants. The predominant causes of preterm delivery were suspected (41.8%) or definite (20%) amniotic infection syndrome, pathological vascular Doppler (25.9%) and preeclampsia/HELLP (9.4%). During the neonatal period, 35 infants (20.6%) developed clinical or blood culture positive EOS, whereas 18 infants (10.6%) suffered from clinical LOS and 27 infants (15.8%) from blood-culture proven LOS. Four patients died during the first 72 h of life.

**Table 1:** Clinical characteristics of the included preterm infants.

Parameters	Frequency (Percentage)
**Multiples**	62 (36.5%)
Gender (male)	93 (54.7%)
**Small for gestational age**	38 (22.4%)
**Reasons for delivery**	
Preterm labor/pROM/AIS	98 (57.6%)
Path. vascular Doppler/path. CTG	44 (25.9%)
Preeclampsia/HELLP	16 (9.4%)
Placental abruption	6 (3.5%)
Others	6 (3.5%)
**Amniotic infection syndrome (AIS)**	
No AIS	65 (38.2%)
Reasonable suspicion of AIS	71 (41.8%)
Definite AIS	34 (20.0%)
**Early-onset-Sepsis (EOS)**	
No EOS	135 (79.4%)
Clinical Sepsis	33 (19.4%)
Blood-culture pos. EOS	2 (1.2%)
**Late-onset-Sepsis (LOS)**	
No LOS	121 (71.2%)
Clinical Sepsis	18 (10.6%)
Blood-culture pos. LOS	27 (15.8%)
Death during the first 72h	4 (2.4%)

AIS, amniotic infection syndrome; SGA, small for gestational age; pPROM, preterm rupture of membranes; CTG, Cardiotocography; HELLP, Hemolysis, elevated liver enzymes, low-platelets syndrome; AIS, amniotic infection syndrome; EOS, early onset sepsis; LOS, late onset sepsis.

### Circulating Gal-1 and Gal-3 Levels Decline After Birth

We analyzed circulating gal-1, -3, and -9 levels during the period of highest infection vulnerability (between day 1 and 28 of life). As shown in **Table 2**, gal-1 and gal-3 levels decreased during the first seven days of life to a plateau while gal-9 peripheral levels in preterm infants did not change remarkably (**Table 2** and **Supplementary Figure 1**). The level of gal-1 and 3 was significantly higher at day 1 as compared to any other postnatal day after the first week of life in single comparison. In multivariable testing for the combined influences of gestational age (GA), birth weight and postnatal age, gestational and postnatal age remained significant for gal-1 (GA: B = -6.54, 95% CI: -9.28/-3.79, p < 0.001; postnatal age: B = -2.22, 95%CI: -2.69/-1.74, p < 0.001) and gal-3 (GA: B = -0.66, 95% CI: -1.06/-0.27, p < 0.001; postnatal age: B = -0.17, 95%CI: -0.26/-0.09, p < 0.001). Interestingly, circulating levels of gal-1 were higher than gal-3 and gal-9.

**Table 2:** Postnatal dynamics of gal-1, -3, and -9 levels during the first 28 days of life in preterm infants.

Galectins fingerprints	Day 1, n = 97	Day 3, n = 22	Day 7, n = 66	Day 14, n = 47	Day 21, = 86	Day 28, n = 24
Gal-1 (ng/ml) (mean/median/SD/p in comparison to day 1)	88.85/70.53/60.27/-	84.60/ 46,09/93,41/n.s.	*32.65/26.21/25.43/<0.001	*29.58/23.84/19.19/<0.001	*27.45/21.78/23.29/<0.001	*23.66/22.49/11.39/0.001
Gal-3 (ng/ml) (mean/median/SD/p in comparison to day 1)	11.36/8.97/9.1/-	8.95/7.56/6.14/n.s.	*5.52/4.67/3.92/<0.001	*7.25/6.76/3.98/0.046	*6.92/6.02/4.46/0.013	*5.73/5.552.45/0.023
Gal-9 (ng/ml) (mean/median/SD/p in comparison to day 1)	3.16/2.62/2.06/-	3.93/3.31/2.66/n.s.	3.23/2.79 1.64/n.s.	3.43/2.28/3.3/n.s.	3.15/2.48/2.76/n.s.	3.93/3.17/3.24/n.s.

*Significant difference in comparison to day 1 (p < 0.05).

### Gal-1 and Gal-3 Levels Inversely Correlate With Gestational Age at Birth

**Figure 1:**
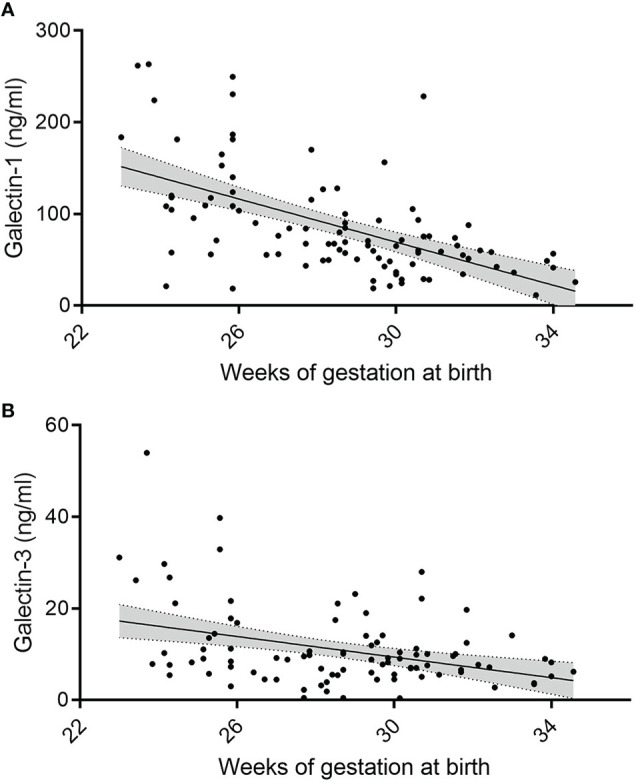
Change in serum gal-1 and gal-3 levels in relation to postnatal and gestational age. **(A, B)** Levels of gal-1 (ng/ml) **(A)** and gal-3 (ng/ml) **(B)** at day 1 correlated to gestational age (in weeks, *P < 0.05*, N = 96) as annalyzed by Mann-Whiteney U-test, respectively.

As outlined in **Figures 1A, B**, concentrations of gal-1 and gal-3 on day 1 and day 3 of life inversely correlate with gestational age (Day 1: gal-1: R^2^ = -0.34, *P* < 0.001, n = 97; gal-3: R^2^ = -0.077, *P* = 0.006, n = 97; Day 3: gal-1: R^2^ = -0.33, *P* = 0.005, n = 22; gal-3: R^2^ = -0.077, *P* = 0.006, n = 22). For birth weight, however, the correlation was less pronounced (Day 1: gal-1: R^2^ = -0.21, *P* < 0.001, n = 97; gal-3: R^2^ = -0.053, *P* = 0.023, n = 97; Day 3: gal-1: not significant; gal-3: R^2^ = -0.19, *P* = 0.023, n = 22). A weak correlation was found between levels of gal-1 and gal-3 on day 21 (gestational age: gal-1: R^2^ = -0.14, *P <* 0.001, n = 86; gal-3: R^2^ = -0.22, *P <* 0.001, n = 86, birth weight: gal-1: R^2^ = -0.21, *P* = 0.001, n = 86; gal-3: R^2^ = -0.08, *P* = 0.007, n = 86).

We found no correlation between gal-9 and gestational age or birth weight. In addition, SGA, gender, and multiple birth had no impact on gal-1, -3, or -9 serum concentration in our cohort.

### Early Inflammation Is Associated With Increased Galectin Levels in Preterm Infants

**Figure 2:**
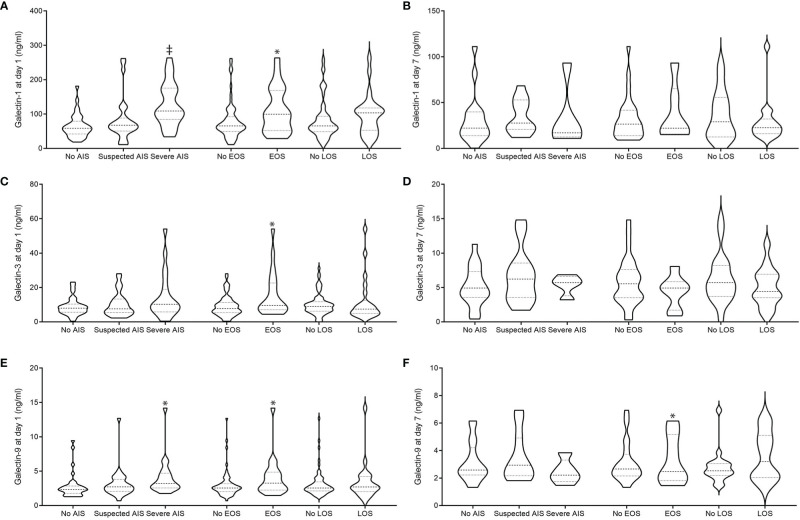
Peripheral blood gal-1 **(A, B)**, gal-3 **(C, D)**, and gal-9 **(E, F)** levels in relation to AIS and EOS at day 1 **(A, C, E)** and 7 **(B, D, F)** of life AIS was diagnosed clinically: definite signs were noted: maternal fever or fetal tachycardia, maternal increase in white blood cell count >10/ml or c-reactive protein levels (> 20 mg/L) without other focus of infection, painful uterus, foul-smelling amniotic fluid, and either preterm labor or preterm rupture of membrane. Suspicion of AIS was diagnosed with one clinical sign. EOS was diagnosed along the NEO-KISS criteria (clinical sepsis or blood-culture proven sepsis). *P < 0.05 and ^‡^P < 0.025 as analyzed by Mann-Whitney U-test (EOS) and Kruskal-Wallis (AIS).

We hypothesized that perinatal inflammation as present in definite or suspected AIS or EOS/LOS may influence systemic gal levels. Using single comparisons, we found higher levels of galectin 1 and 9 in infants born to mothers with severe AIS (n = 23) during the first day of life (**Figure 2**) compared to infants born to mothers with suspected (n = 37) or without AIS (n = 37). At day 1 of life, for example, gal-1 levels were remarkably increased in infants born to severe AIS (**Figure 2A**; mean ± SD; severe AIS: 128.5 ng/ml ± 61.5, suspected AIS: 89.5 ng/ml ± 69.3, no AIS: 63.5 ± 34.9; *P <* 0.001), which was also observed for gal-9 (**Figure 2E**; severe AIS: 4 ng/ml ± 1.6, suspected AIS: 3 ng/ml ± 1.9, no AIS: 2.8 ± 1.8; P = 0.011). This difference was not evident for gal-3 and on day 3 of life (**Tables 2B, D, F**).

We found that infants with clinical or blood-culture proven early-onset sepsis (EOS, n = 24) had higher levels of all galectins at day 1 of life compared to unaffected preterm infants (no EOS, n = 73); (gal-1 (ng/ml) EOS: 112.9 ± 101.7 versus no EOS: 80.9 ± 65.4; *P* = 0.018, **Figure 2A**; gal-3 (ng/ml) EOS: 15.2 ± 12.2 versus no EOS: 10.1 ± 7.5, *P* = 0.036, **Figure 2C**; gal-9 (ng/ml) EOS: 3.8 ± 2.9 versus no EOS: 2.9 ± 2.5; *P* = 0.049, **Figure 2E**). At day 7 of life, we found gal-9 to be increased in preterm infants suffering from early-onset sepsis [gal-9 (ng/ml) EOS: n = 16, 4.3 ± 2.3 versus no EOS: n = 50, 2.9 ± 1.2; *P <* 0.028].

Galectin levels did not differ remarkably in infants who developed LOS as compared to unaffected infants.

### Correlation Between Gestational Age, Postnatal Age, and Inflammation

To test the hypothesis that galectins levels at different postnatal time points are significantly associated with clinical parameters, we applied the GEE statistical model. AIS (B =4.61, p = 0.003) was associated with the dynamics of gal-1 levels. Gestational age (gal-1: B = 0.22, p = 0.015) was not significant associated with the dynamics of any galectin level after adjustment for multiple testing.

## Discussion

To our best knowledge, we herein present the first observational data of gal-1, -3, and -9 levels in a cohort of preterm infants. We describe that gal-1 and -9 levels are elevated in infants born in an inflammatory milieu. Increased levels of all galectins are found to be associated with early-onset sepsis. Gal-1 and -3 levels are inversely correlated with gestational age and rapidly decline during the neonatal period.

The role of galectins during amniotic infection is not yet understood. At term, healthy laboring women have diminished decidual gal-1 and gal-3 expression levels as compared with non-laboring women (7). Our data indicate that increased galectin levels in preterm deliveries may be involved in the pathophysiological mechanisms of amniotic infection locally at the fetomaternal interface (13) but also in the systemic immune response of the offspring. We hereby hypothesize that galectins may directly or indirectly interact with pathogens (of early-onset infection) and thereby regulate immune responses.

Noteworthy, we found an inverse correlation between levels of gal-1 and gal-3 in the newborn and gestational age at delivery. This observation is in contrast with our previous studies showing a positive correlation of gal-3 concentrations with gestational age in neonatal whole cord blood (15), which may be related either to a different kinetics of galectin regulation in cord versus peripheral blood or to differences in the experimental design of both studies, as early onset sepsis cases were excluded from our previous analyses. In comparison with the increasing number of studies focusing on the galectin profile in the maternal circulation, information on the systemic variations of galectin levels in the fetus/newborn is very scarce to date. Current evidence indicates that uneventful pregnancy is associated with a steady increase of maternal circulating levels of the three galectins investigated in this study from the first to the third trimester (10, 22, 23), suggesting that the kinetics of galectin secretion may be subject to regulation by different sets of signals operating in the maternal and fetal compartment. Thus, one possibility that requires further investigation is that galectins function to shape innate immunity until the newborn achieves full immune competence and that the very same signals that drive fetal maturation and trigger parturition are also involved in the regulation of the systemic galectin signature as a mechanism to prepare the fetus for the challenges of extrauterine life. In support of this notion is the finding that hormones of the HPA axis involved in the signaling cascade driving parturition [i.e., CRH, urocortins (24), glucocorticoids (25–28), DHEA sulfate (29)] have been shown to modulate galectin expression in a variety of pathophysiological settings and cell types including innate imune mediators. On the other hand, since earlier preterm births are more frequently associated with infections and activation of a fetal inflammatory response syndrome (i.e., chorioamnioninitis) (30), it is also possible that elevated galectin levels at earlier gestational ages are due to the described function of galectins as damage associated molecular patterns (DAMPs), acting as ‘alarmin-like’ molecules to signal tissue damage and promote an effector response from immune cells (31–33). In this context, an interesting observation is that pregnancies with preterm birth due to infection show significantly higher levels of CRH, urocortin 2, and CRH-R1 in comparison with patients with idiopathic preterm birth or premature rupture of membranes without infection (34), suggesting that placental expression of stress-related pathways is activated in infective processes.

Our study further revealed an association between elevated levels of circulating galectins and AIS/EOS in premature infants, with gal-9 emerging as a possible biomarker for early onset neonatal sepsis. Considering that, in contrast to the late onset form, EOS is exclusively caused by vertical transmission of bacterial infection in utero or intrapartum (35), our results point to a specific role played by galectins in modulating the fetal immune response against intraamniotic infection. Accordingly, we have previously demonstrated that *in vitro* stimulation with an invasive strain of *S. agalactiae* (a Group B Streptococcus commonly associated with EOS) induced gal-3 expression in cord blood samples of healthy neonates (15). The role of gal-3 in inflammation and infection has been well established, serving mainly a pro-inflammatory function: gal-3 has been shown to enhance neutrophil adhesion and macrophage migration, promotes their oxidative burst and functions as a chemoattractant for monocytes and macrophages (36–39), acting as an important modulator of innate immunity. Being the first line of defense, the innate immune system is of outmost importance for protection against infection in the perinatal period and thus these features of gal-3 make it a paramount mediator driving neonatal immunity in both term and preterm infants. In this regard, it has been shown that circulating levels of gal-3 are higher in neonates than in the adult, and that resting neonatal neutrophils exhibit a phenotype of hyperresponsiveness to gal-3 that is further enhanced in births by vaginal delivery in comparison to C-sections (40). Interestingly, cord blood levels of galectin 3 binding protein (Gal3BP, an immunoregulatory glycoprotein with affinity for gal-1 and gal-3) at birth are increased in preterm neonates and also show an inverse correlation with gestational age, whereas decreased levels of the protein were associated with fetal infections (i.e., choriamnionitis) (17); which led to the assumption that circulating Gal3BP may complement and modify gal-3 activity in the neonate. Further, recent studies have shown that mice deficient for *Lgals3BP* display enhanced LPS-induced proinflammatory cytokine release and increased sensitivity to LPS-induced endotoxin shock (41). In view of these findings, we speculate that impaired galectin regulation may contribute to the increased susceptibility of preterm infants to infection as opposed to term neonates or adults. However, the differences in the galectin levels may also be a result of infection instead of its cause. Approaches aimed at promoting gal-3 expression in neonates might, in future, contribute to novel therapeutic protocols for management of perinatal infection. Further studies should be encouraged to elucidate the functional relevance of our findings and to address whether galectins mediate inflammation-induced preterm birth and their potential application as a target for clinical trials.

## Data Availability Statement

The raw data supporting the conclusions of this article will be made available by the authors, without undue reservation.

## Ethics Statement

The studies involving human participants were reviewed and approved by University of Lübeck Ethical Committee (IRON AZ 15-304). Written informed consent to participate in this study was provided by the participants’ legal guardian/next of kin.

## Author Contributions

SMB and CH designed the study and secured grant funding. KF, CH, NF, GB, and SMB performed the experiments and/or analyzed the data. KF, CH, and SMB wrote the manuscript. All authors contributed to the article and approved the submitted version.

## Funding

This study was supported by the German Ministry of Research and Education (BMBF; Clinical Leave stipend of the German Centre for Infection Research to KF), grants from the Deutsche Forschungsgemeinschaft (German Research Foundation) to CH (HA6409/5-1) and to SMB (BL1115/2-1, BL1115/4-1 and Heisenberg program BL1115/7-1).

## Conflict of Interest

The authors declare that the research was conducted in the absence of any commercial or financial relationships that could be construed as a potential conflict of interest.

## Supplementary Material

Supplementary Figure 1 Levels of gal-9 (ng/ml) at day 1 correlated to gestational age (in weeks, *P < 0.05*, N = 96).
