# Supplementary figures and images for: Galectin-Levels Are Elevated in Infants Born Preterm Due to Amniotic Infection and Rapidly Decline in the Neonatal Period
Source: Front Immunol. 2021 Feb 25;11:599104. doi: 10.3389/fimmu.2020.599104 (PMC7949913; doi:10.3389/fimmu.2020.599104)

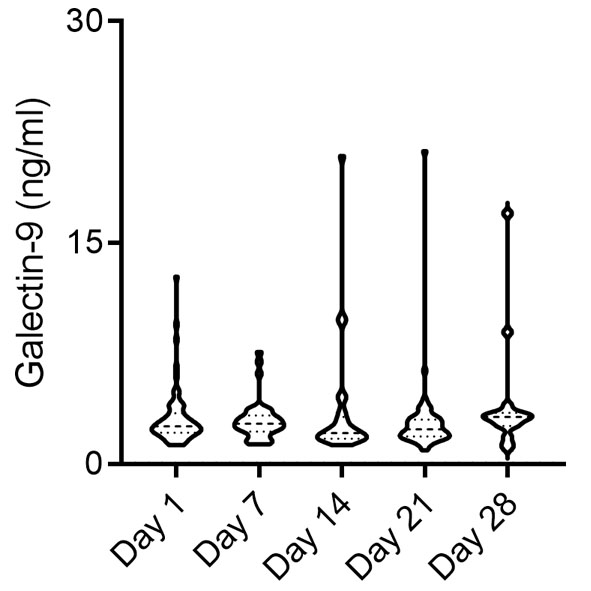

Supplement: Supplementary Figure 1 — Levels of gal-9 (ng/ml) at day 1 correlated to gestational age (in weeks, P < 0.05, N = 96). [file Image_1.jpeg]
