# Supplementary material for: Galectin-Levels Are Elevated in Infants Born Preterm Due to Amniotic Infection and Rapidly Decline in the Neonatal Period
Source: Front Immunol. 2021 Feb 25;11:599104. doi: 10.3389/fimmu.2020.599104 (PMC7949913; doi:10.3389/fimmu.2020.599104)
Supplement: Supplementary file 2 [file Table_1.docx]

**Suppl. Table 1.** Inflammatory details of the included preterm infants. AIS: amniotic infection syndrome, EOS early onset sepsis, LOS late onset sepsis

|  | | AIS | | | EOS | | | LOS | | |
| --- | --- | --- | --- | --- | --- | --- | --- | --- | --- | --- |
|  |  | **NO AIS** | **Reso-nable suspi-cion** | **Defi-nite AIS** | **No EOS** | **Clinical Sepsis** | **Blood culture pos. EOS** | **No LOS** | **Clinical Sepsis** | **Blood culture pos. LOS** |
| Total number  (frequency/ percentage) | | 65 /  38.2% | 71 /  41.8% | 34 /  20% | 135 / 79.4% | 33 / 19.4% | 2 / 1.2% | 121 / 71.2% | 18 / 10,6% | 27 / 15,8% |
| Number of samples for each timepoint  (day 1/3/7/ 14/21/28) | | 37/8/ 27/18/32/6 | 37/11/25/15/36/12 | 23/3/ 14/14/18/6 | 73/18/50/60/71/20 | 22/4/16/6/13/4 | 2/0/0/1/1/0 | 68/14/41/33/60/17 | 16/3/ 11/17/6/5 | 13/5/ 14/7/ 19/2 |
| AIS | **NO AIS** | 65 | - | - | 56 | 9 | 0 | 45 | 8 | 11 |
|  | **Resonable suspicion** | - | 71 | - | 62 | 9 | 0 | 56 | 5 | 9 |
|  | **Definite AIS** | - | - | 34 | 17 | 15 | 2 | 20 | 5 | 7 |
| EOS | **No EOS** | 56 | 62 | 17 | 135 | - | - | 100 | 12 | 20 |
|  | **Clinical Sepsis** | 9 | 9 | 15 | - | 33 | - | 21 | 6 | 6 |
|  | **Blood culture pos. EOS** | 0 | 0 | 2 | - | - | 2 | 0 | 0 | 1 |

**Suppl. Table 2.** Results of the generalized estimating equation (GEE) models to determine the association between gal-1 levels on different postnatal timepoints with clinical parameters.

Interaction effects of day of life and AIS, day of life and EOS, day of life and LOS, day of life and gestational age were tested (AIS: amniotic infection syndrome; EOS: early-onset sepsis; LOS: late-onset sepsis; GA: gestational age, day: day of life). P<0.01 (Bonferroni-correction)

|  | Estimate (B) | SE | 95% CI | | p-value |
| --- | --- | --- | --- | --- | --- |
| Gestational age (GA) | -6.74 | 1.63 | -9.94 | -3.54 | <0.001 |
| Day of life (day) | -8.32 | 2.42 | -13.06 | -3.57 | <0.001 |
| No AIS | -54.525 | 21.2081 | -96.092 | -12.958 | .010 |
| Severe AIS | 0.978 | 20.7011 | -41.551 | 39.596 | .962 |
| EOS | 9,926 | 16.2918 | -22.006 | 41.857 | .542 |
| Interactions | | | | | |
| Day x GA | 0.22 | 0.09 | 0.05 | 0.39 | **0.015** |
| Day x no AIS | 4.608 | 1.5349 | 1.600 | 7.617 | **.003** |
| Day x severe AIS | 1.236 | 1.5750 | -1.851 | 4.322 | .433 |
| Day x EOS | 0.213 | 1.3252 | -2.384 | 2.810 | .872 |
